# Recent insights into cotton functional genomics: progress and future perspectives

**DOI:** 10.1111/pbi.12856

**Published:** 2018-01-15

**Authors:** Javaria Ashraf, Dongyun Zuo, Qiaolian Wang, Waqas Malik, Youping Zhang, Muhammad Ali Abid, Hailiang Cheng, Qiuhong Yang, Guoli Song

**Affiliations:** ^1^ State Key Laboratory of Cotton Biology Institute of Cotton Research Chinese Academy of Agricultural Sciences Anyang Henan China; ^2^ Genomics Lab Department of Plant Breeding and Genetics Faculty of Agricultural Sciences and Technology Bahauddin Zakariya University Multan Punjab Pakistan

**Keywords:** genome sequencing, cotton databases, gene discovery tools, CRISPR/Cas9, cotton improvement, biotic and abiotic stresses

## Abstract

Functional genomics has transformed from futuristic concept to well‐established scientific discipline during the last decade. Cotton functional genomics promise to enhance the understanding of fundamental plant biology to systematically exploit genetic resources for the improvement of cotton fibre quality and yield, as well as utilization of genetic information for germplasm improvement. However, determining the cotton gene functions is a much more challenging task, which has not progressed at a rapid pace. This article presents a comprehensive overview of the recent tools and resources available with the major advances in cotton functional genomics to develop elite cotton genotypes. This effort ultimately helps to filter a subset of genes that can be used to assemble a final list of candidate genes that could be employed in future novel cotton breeding programme. We argue that next stage of cotton functional genomics requires the draft genomes refinement, re‐sequencing broad diversity panels with the development of high‐throughput functional genomics tools and integrating multidisciplinary approaches in upcoming cotton improvement programmes.

## Introduction

Cotton (*Gossypium hirsutum*) is a foundation of the global economy, prized for its important renewable fibre resource. It serves as an ideal plant for different biological studies such as genome evolution, polyploidization and single‐celled biological processes (Qin and Zhu, 2011; Shan *et al*., 2014). Decoding cotton's genome provides useful understanding about the agronomic and functional importance of polyploidy and genome size variations within the genus *Gossypium* (Chen *et al*., 2007). However, evolution and function of allopolyploid cotton genome is complicated by the presence of two subgenomes (*A*_*T*_ and *D*_*T*_) in its nucleus. About 5–10 million years ago (MYA), the African‐derived ‘A’ diploid genome diverged from the eudicot progenitor simultaneously with the diploid genome ‘D’ which was native to Mexican (Wendel, 1989; Wendel and Albert, 1992). Then around 1–2 MYA, these two species were reunited together by the transoceanic dispersal of an A‐genome ancestor (*Gossypium arboreum*) to the New World and hybridized with a D‐genome ancestor (*Gossypium raimondii*) followed by chromosome doubling, which produced the allotetraploid cotton (Wendel, 1989). These well‐established relationships between the allotetraploid and diploid cotton genomes help us to explore the evolution of gene expression, because most of the gene functions are highly conserved between wild as well as diploid and tetraploid cotton species.

Whole‐genome sequencing is a fundamental component for comprehensive molecular analysis, and for several thousands of plant species, genome sequencing projects are now complete or underway. Compared with model plants, that is Arabidopsis, rice and maize, the whole‐genome sequencing of cotton was lacking behind. During the last decade, sequenced genomes of tetraploid cotton (Li *et al*., 2015; Liu *et al*., 2015; Yuan *et al*., 2015; Zhang *et al*., 2015b) and their diploid progenitors (Li *et al*., 2014b; Paterson *et al*., 2012; Wang *et al*., 2012) have been released that provide critical understanding of the evolution and differentiation of genome structures. Though, knowledge of the precise sequences and position of all the genes of an organism is an initial step to explore how biological systems work together. Previously, various studies have performed to compare the structural variations in genomes, which showed the differences in the expression pattern rather than in the absence and presence of genes (Gingle *et al*., 2006). In this respect, functional genomics is the main approach which is generally referred as ‘development and application of global experimental approaches to evaluate gene functions by using the information and reagents obtained from structural genomics’. It helps us to understand the basic plant biology and exploit the genomic information for cotton improvement, which is a vital step for manipulating cotton genes in agriculture. However, in cotton functional genomics, a persistent challenge is the absence of genetic and molecular tools partly due to large genome size, low transformation efficiency and long growth cycle. In this article, we provide an over‐review of the currently available tools and resources for cotton functional genomics with its recent advances for different important traits. Ultimately, this overview helps in assembling a final list of candidate genes that might be employed in future novel cotton breeding programme.

## Tools and resources for cotton functional genomics

**Figure 1:**
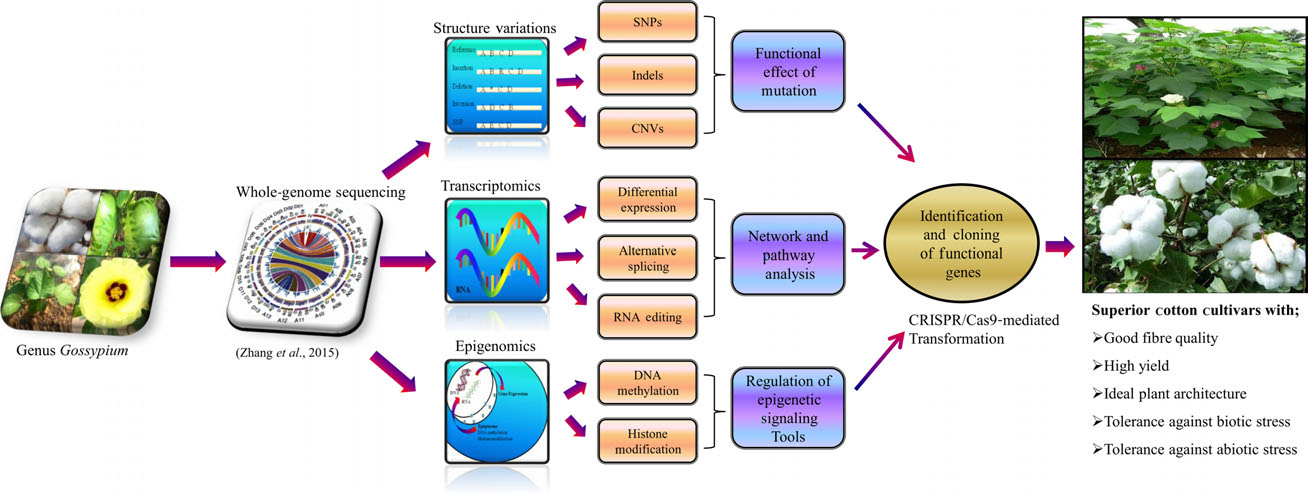
A scheme of the sequential research processes from whole‐genome sequencing to practical functional genomics in cotton. This figure shows the integrative approach of comprehensive information flows from the whole‐genome sequencing to practical functional genomics in cotton. It systematically represents the typical data evaluation path with bioinformatics tools in genomics, transcriptomics and epigenomics technologies to analyse the genomic mutations, differential gene expression and regulation of epigenetic signalling tools. It also incorporates protein expression data into appropriate genes and functional networks which ultimately facilitate the identification and cloning of functional genes. SNPs (single nucleotide polymorphisms), Indels (insertion/deletion) and CNVs (copy number variations)

Cotton has become a system of choice for functional genomics studies. Here, we overview the available resources and tools for functional genomics studies in cotton and also discuss the ways (Figure 1) in which existing resources or tools can be used to further support large‐scale functional studies in cotton.

## Cotton genome sequencing: progress and implications

Efforts towards increased efficiency of functional genomics are demonstrated by the advancements that initiated from genome sequencing. In the last 20 years, rapid and impressive progress has been made in developing genetically modified cotton cultivars against resistance to insects and herbicides (Guo *et al*., 2015b; Yu *et al*., 2016). Conversely, slow advances have been made in genetic improvements of cotton for plant architecture, flowering, fibre quality, yield and resistance against biotic and abiotic stresses. The successful implementation and accessibility of well‐established whole‐genome sequences of Arabidopsis and rice has facilitated the consortium‐based cotton genome research. In 2007, the Cotton Genome Consortium (Chen *et al*., 2007) set a strategic plan to sequence cotton genomes that they primarily target less‐complicated diploid genomes that can be directly applied to the tetraploid cotton. For the persistent objective of cotton genome sequencing, the D‐genome species *G. raimondii* was prioritized for complete sequencing. A major initial source of cotton genome sequencing was released in 2012 by two independent studies that released the draft genome sequence of *G. raimondii* (Paterson *et al*., 2012; Wang *et al*., 2012), which was a rational step to characterize the larger ‘A’ diploid and ‘AD’ tetraploid cotton genomes.

Two years later, the same research group sequenced the 1694‐Mb genome of *G. arboreum*, which is a supposed donor species for the A chromosome group (Li *et al*., 2014b) in tetraploid cotton. However, the genomes of two existing progenitors (*G. raimondii* and *G. arboreum*) have been sequenced; the precise species that directs the development of the tetraploid cotton species about 1–2 MYA currently not exists (Wendel, 1989). Further, *G. hirsutum* revealed important variations in plant morphology and economic characteristics as compared to diploid cotton species, showing that precise natural and artificial selection has happened during evolution. Therefore, it was essential to sequence the allotetraploid species of cotton to understand the evolutionary history and gain insights into fibre biology. Using the genome sequences of A and D progenitor species, Li *et al*. (2015) and Zhang *et al*. (2015b) simultaneously but independently sequenced the genome of allotetraploid *G. hirsutum*. Besides *G. hirsutum*, Sea Island cotton (*G. barbadense*) is prized due to its superior quality and extra‐long fibre for the fabrication of high‐quality textiles. Considering its importance, genome of the *G. barbadense* was sequenced (Liu *et al*., 2015; Yuan *et al*., 2015), which covered 2470 and 2570 Mb of the genome, respectively.

**Figure 2:**
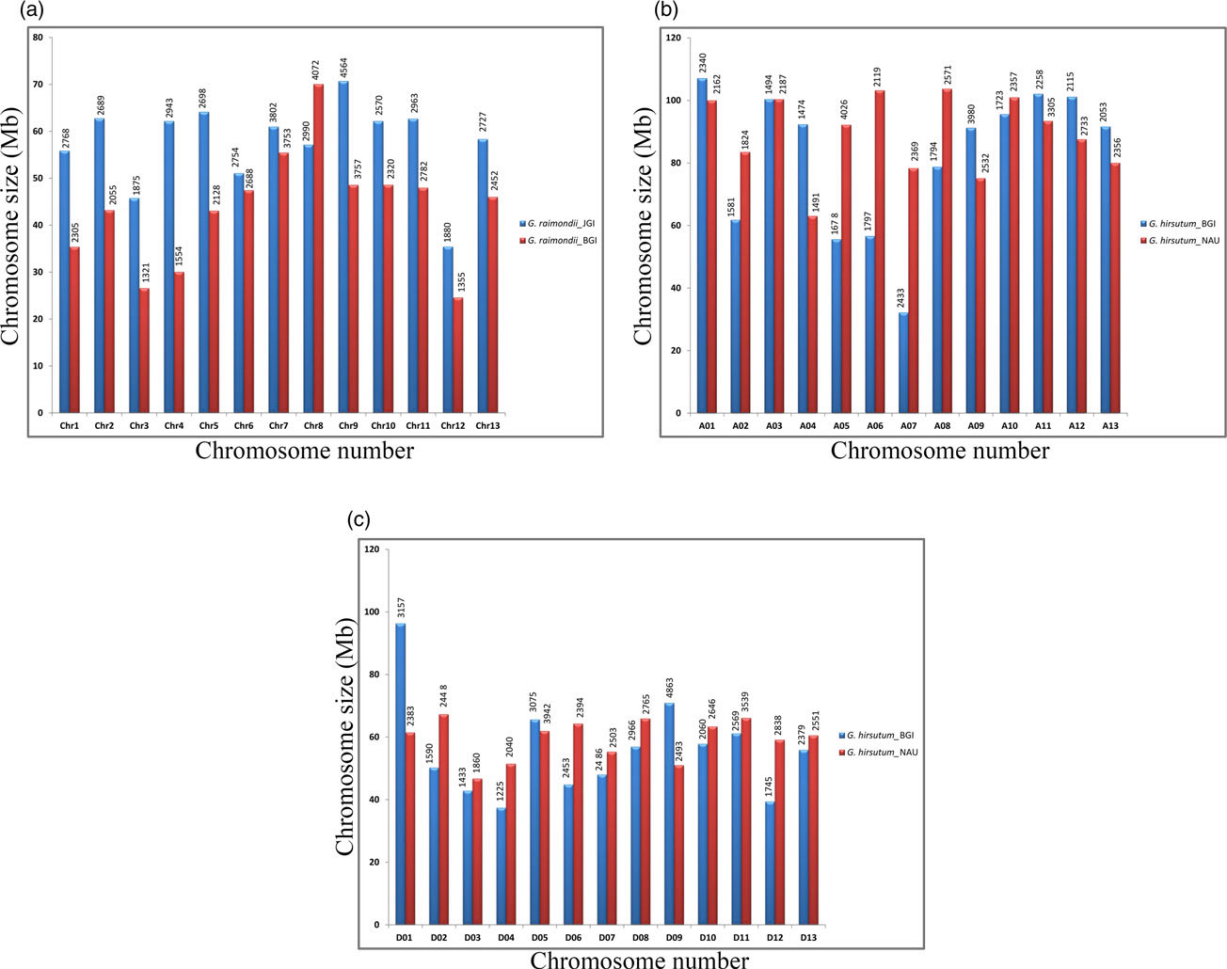
Chromosome size distribution (*y*‐axis) and number of annotated genes (above each bar) of different *Gossypium* species. Differences in chromosome size and number of annotated genes (above each bar) by two independent studies between the sequenced genomes of (a) *G. raimondii* (Paterson *et al*., 2012 (blue); Wang *et al*., 2012 (red)); (b) *At*‐subgenome of *G. hirsutum* (Li *et al*., 2015 (blue); Zhang *et al*., 2015b (red)), and (c) *Dt*‐subgenome of *G. hirsutum* (Li *et al*., 2015 (blue); Zhang *et al*., 2015b (red)). These differences might be due to errors in their assemblies, which in turn also affects the various genome analyses among different cotton species. Currently, we need to devote more efforts in capturing, evaluating and fixing their misassemblies by developing quality control standards.

At present, reference genome sequences for diploid and tetraploid cotton species are released by different groups, but the researchers assumed that some of these sequenced genomes contain assembly errors. For example, differences have been observed in the sequenced and assembled draft genomes of the *G. raimondii* (Paterson *et al*., 2012; Wang *et al*., 2012) and *G. hirsutum* (Li *et al*., 2015; Zhang *et al*., 2015b) by two independent groups in terms of chromosome length and their annotated genes (Figure 2a–c). These differences might be due to errors in their assemblies (Zhang *et al*., 2015b), at least at large scale, which in turn also affects the tremendous amount of genome analysis among different cotton species. Currently, we need to devote more effort in capturing these genome assemblies with a more sceptical eye for careful comparison, evaluation and fixing their misassemblies by developing quality control standards. In addition, re‐sequencing the genome for which there is a reference genome available permits exploration of the association between sequence variations. Recent comprehensive genome assessment by genome‐wide re‐sequencing of 34 (Page *et al*., 2016), 318 (Fang *et al*., 2017b), 147 (Fang *et al*., 2017a) and 352 (Wang *et al*., 2017b) cotton accessions represented extensive collections in order to identify genome regions that are signature of selection. These studies provide new genomic resources that significantly advance molecular breeding in cotton. Particularly, under the guidance of sequence information, the favourable genes that are linked with high yield, wide adaptation and fibre quality can be introgressed between different gene pools to further improve cotton production.

## Gene discovery tools recently available

Sequenced and re‐sequenced cotton genomes are simply the foundation; the main challenge is to discover the features of the genome that explain the biology. The next stage of cotton genomics will entirely expose these biologically active states of DNA, as has been made for other model crop plants where high‐density genetic and fine maps, SNP array platforms, epigenetic modifications and transcript abundance are studied across multiple species and tissues. Previous to the release of the draft genome sequences of four *Gossypium* species, limited ultra‐precision genetic maps were the main obstruction that prevented the intense genetic research and breeding of improved cotton cultivars. Currently, few comparatively dense linkage maps of cotton are available (Guo *et al*., 2008; John *et al*., 2012; Li *et al*., 2016; Wang *et al*., 2015), which provide a platform for high‐throughput marker development, gene mapping, gene isolation and cloning. Moreover, during the last decade, at least 1075 QTLs from 58 studies of intraspecific *G. hirsutum* and 1059 QTLs from interspecific populations of *G. hirsutum* × *G. barbadense* have been published for fibre quality, yield, seed quality and resistance against biotic and abiotic stresses (Said *et al*., 2015). However, these identified QTLs reside at large genomic regions that may contain several genes, provide only coarse resolution for marker‐assisted selection. Therefore, it is also crucial to fine map the genomic regions with large number of markers that will enhance the efficiency of selection, which ultimately helps to clone the genes present at the target loci. In cotton, fine mapping of few important genes and QTLs has been reported, that is fine mapping of glandless gene (Cheng *et al*., 2016b), leaf shape (Andres *et al*., 2014) and fibre quality‐related QTLs (Fang *et al*., 2017c; Liu *et al*., 2016a; Xu *et al*., 2017).

The rising efficacy of NGS technique and advanced *in silico* methods has permitted the development of single nucleotide polymorphisms (SNPs) at the whole‐genome level, even for the 2.5‐Gb genome of allotetraploid cotton. In cotton, SNP63K has been developed that contains assays for 45 104 and 17 954 putative intraspecific and interspecific SNP markers (Ashrafi *et al*., 2015). This initial effort for developing SNP63K array of cotton provides a standard high‐throughput genotyping tool and a base for the genetic analysis of economically and agronomically important traits. As a large proportion of the genome was affected by copy number variations (CNVs) rather than SNPs, they may help to explore several phenotypic variations that are not captured by SNPs. Many evidences support that CNVs are prevalent in plant genomes that can change gene structure, dosage and gene regulation (Mills *et al*., 2011), and mainly CNV‐affected genes are related to important traits. In cotton, 989 CNV‐affected genes have been identified (Fang *et al*., 2017b), which are related to cell wall organization, plant type and translational regulation.

Recently, transcriptome profiling has evolved into the most important tool demonstrating how information obtained from sequence data can be transformed into an extensive knowledge of gene function. In this regard, RNA‐Seq has revealed strong potential for whole‐genome transcriptome profiling as it allows the direct sequencing of transcripts by high‐throughput sequencing technologies. The recent transcriptome assembly of the *G. hirsutum* inbred line TM‐1 together with an assembly of all publicly available ESTs (expressed sequence tags) (Ashrafi *et al*., 2015) served as a reference for RNA‐Seq‐based SNP identification of cotton. Also, the application of the diploid and tetraploid genome sequence and NGS technology to practise RNA‐Seq analysis of large‐scale gene expression in cotton has been published by many reports. For instance, transcriptome analysis for leaf senescence (Lin *et al*., 2015), fibre development (Islam *et al*., 2016a; Naoumkina *et al*., 2015; Yoo and Wendel, 2014), biotic stress (Artico *et al*., 2014; Xu *et al*., 2011) and abiotic stress (Bowman *et al*., 2013; Zhang *et al*., 2016a) has been reported. However, RNA‐Seq technique faces some challenges such as library construction, development of efficient methods to store and process large amounts of data (Wang *et al*., 2009). It is anticipated that once these obstacles to the extensive use of RNA‐Seq are overcome, this technique will become the major tool for transcriptome analysis (Zhao *et al*., 2014).

Besides genetic factors, many traits in living things are controlled by other processes known as epigenetic modifications that resolve whether, when and how many genes are expressed. There are many epigenetic signalling tools that controlled gene expression, but most common is the DNA methylation (Phillips, 2008), which has appeared to play an important role in evolution and morphological diversity in crop plants (Cubas *et al*., 1999; Suzuki and Bird, 2008). In cotton, DNA methylation changes are related to seasonal variation in the development of fibres (Jin *et al*., 2013) and different tissues (Osabe *et al*., 2014). For example, dynamic role of methylation in ovule and fibre development showed that RdDM (RNA‐directed DNA methylation)‐dependent CHH methylation is related to gene activation in ovules, while CMT2 (chromomethylase2)‐dependent methylation guides to the silencing of genes in fibres (Song *et al*., 2015). Subsequently, 519 cotton genes are epigenetically modified between wild and domesticated cotton cultivars, some of which are related to agronomic and domesticated traits (Song *et al*., 2017). This finding gives insights into epigenetic regulation for the development of different traits and polyploid evolution of cotton. So, knowing how the methylome changed during evolution and domestication helps to bring this technology one step closer to reality.

## Functional genomics databases for cotton

A comprehensive study of any genome depends on the availability of information regarding genome sequence, map position, mRNA and protein expression, metabolism and allelic variation. Hence, with the development of enormous omics data sets, it is important to have functional genomics database that permits users to easily obtain and visualize genomic information. Currently, many functional genomics databases are available for the cotton research community: the CottonGen (https://www.cottongen.org), Cotton Functional Genomic Database (CottonFGD; https://cottonfgd.org), Cotton Genome Resource Database (CGRD; http://cgrd.hzau.edu.cn/index.php), Cotton Genome Database (CottonDB; http://www.cottondb.org), Cotton Genome Project (CGP; http://cgp.genomics.org.cn/page/species/index.jsp), Platform of Functional Genomics Analysis in *Gossypium raimondii* (GraP; http://structuralbiology.cau.edu.cn/GraP/about.html), Comparative Evolutionary Genomics of Cotton (http://cottonevolution.info/), Join Genome Institute (JGI; http://jgi.doe.gov) and Database for Co‐expression Networks with Function Modules (ccNET; http://structuralbiology.cau.edu.cn/gossypium/).

CottonGen is the most important curated web‐based intellectual database, offering easy access to available genomic and genetic data of cotton. It contains annotated whole‐genome sequences of different cotton species, unigenes from ESTs, genetic maps, markers trait loci, genes and germplasm resources. Similarly, CottonFGD also provides a quick and easy access to genome sequences, functional annotations, transcriptome and genome re‐sequencing data for all of the sequenced *Gossypium* genomes. However, ccNET displays co‐expression networks and identified functional modules from diploid and polyploid cotton species including 1155 and 1884 modules in *G. arboreum* and *G. hirsutum*, respectively.

## Potential of CRISPR/Cas9 in cotton gene editing

Clustered regularly interspaced short palindromic repeat (CRISPR)‐associated protein 9 (Cas9) from *Streptococcus* pyogenes (Sapranauskas *et al*., 2011) is a fast developing genome editing technology that has been effectively employed in many model plants (Belhaj *et al*., 2015). A distinctive feature of CRISPR/Cas9 is that DNA cleavage sites are recognized through Watson–Crick base pairing (Lowder *et al*., 2015) by three components: Cas9 protein, CRISPR‐RNA (crRNA) and trans‐activating crRNA (trancrRNA) (Karvelis *et al*., 2013; Mei *et al*., 2016). The utilization of the CRISPR/Cas9 system as a genome engineering tool came out when it was revealed that the target DNA sequence could be simply re‐programmed by altering 20 nucleotides in the CRISPR‐RNA (Jinek *et al*., 2012). Further, multiple gRNAs with diverse sequences could also be used to get multiplex genome engineering at various loci at the same time. This milestone study established that the CRISPR/Cas9 is a simple, efficient, economical and multipurpose tool for gene mutation, gene expression repression or activation and genome editing.

In plant biology, the first application of CRISPR/Cas9‐based genome editing (Li *et al*., 2013c; Shan *et al*., 2013) demonstrated its vast adaptability in the model species Arabidopsis as well as in crop plant rice. Subsequently, it has been applied in other crop plants, that is maize (Liang *et al*., 2014) and wheat (Wang *et al*., 2014b). In cotton, the application of CRISPR/Cas9 is still at its infancy. Most recently, multiple sites genome editing through CRISPR/Cas9 system in allotetraploid cotton by targeting arginase (*GhARG*), discosoma red fluorescent protein2 (*DsRed2*) and chloroplast development (*GhCLA1*) genes proves that it is highly reliable and effective for cotton genome editing (Wang *et al*., 2017c,2017d). It is expected that the potential and applications of CRISPR/Cas9 in cotton genome editing are certain to be further developed over time. In future, improvements will continue to increase its use from mutant generation to accurate gene regulation at noncoding enhancer regions in cotton.

## Functional genomics for different traits

**Figure 3:**
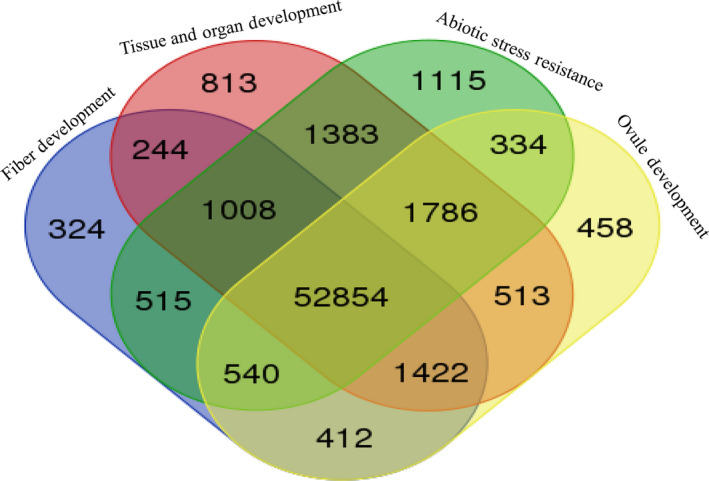
A venn diagram of the differentially expressed genes during fibre development, tissue and organ development, abiotic stress resistance and ovule development. The functional distribution of annotated genes from *G. hirsutum* (Zhang *et al*., 2015b) highlights that 52 854 differentially expressed genes were commonly identified during fibre, organ, tissue and ovule development and resistance against abiotic stress. However, more genes were differentially expressed during stress resistance than during fibre and organ development.

With the success of whole‐genome sequencing of cotton, its annotated genes were assigned some degree of functions by comparing them with the sequences of genes with known function and RNA‐Seq analysis. For example, the functional allocation of *G. hirsutum* genes (Zhang *et al*., 2015b) was shown by a Venn diagram. The RNA‐Seq data in fragments per kilobase of exon per million fragments mapped (FPKM) of each *G. hirsutum* gene were downloaded from CottonFGD. The venn diagram was constructed by online analysis tool (http://bioinformatics.psb.ugent.be/webtools/Venn/). The distributions of these genes (Figure 3) highlight that 52 854 differentially expressed genes were commonly identified during fibre, organ and ovule development and resistance against abiotic stress. Interestingly, there were several more differentially expressed genes during the stress resistance (1115) than during fibre and organ development, implying that the stress resistance is more complicated transcript regulation. However, knowing gene functions by comparative analysis and RNA‐Seq mainly does not give an insight into their specific role. In this regard, large‐scale functional genomics mainly helps in which all of the genes will be assigned functions on the basis of experimental verification.

## Fibre quality

Cotton fibres are single‐celled trichomes that originate from outer integument cells of the ovular surface. The cotton fibre undergoes a complex developmental programme that can be divided into four overlapping stages: fibre cell initiation, elongation, secondary cell wall biosynthesis and maturation (Manik and Ravikesavan, 2009; Wilkins and Jernstedt, 1999). Fibre initiation occurs around the time of anthesis during which about 30% of fibre primordia differentiate into mature fibres (Tiwari and Wilkins, 1995), while cell exhibits highly emphasized polarized expansion during fibre elongation (0–25 day postanthesis) (John and Keller, 1996). The secondary cell wall is primarily comprised of cellulose, which generally occurs between 24 and 27 day postanthesis (Wilkins and Jernstedt, 1999). The last stage of fibre development, maturation is related to the accumulation of minerals and a concurrent decrease in water potential (John and Keller, 1996). Although the above‐mentioned developmental stages are coinciding, each stage has its own specific features of physiological and cellular states. The genetic complexity of the cotton fibre transcriptome lies in the involvement of ~18 000 and 36 000 genes in diploid and allotetraploid cotton genomes (Arpat *et al*., 2004).

Currently, cotton fibre has become a trait of primary interest and several efforts have been made (Table 1) to identify fibre‐related genes and their functions to improve fibre quality such as *E6* (John and Crow, 1992), *GhExp1* (Harmer *et al*., 2002), *GhSusA1* (Jiang *et al*., 2012), *PIP2s* (Li *et al*., 2013b) and *GA20ox* (Bai *et al*., 2014). Additionally, actin cytoskeleton (Li *et al*., 2005), polysaccharide biosynthesis, signal transduction and protein translocation (Sun *et al*., 2017)‐related genes are also preferentially expressed in various fibre developmental pathways. Among these cotton fibre genes, some are predominantly expressed during fibre initiation (Deng *et al*., 2012; Hu *et al*., 2016), some are highly expressed during secondary cell wall biosynthesis (Brill *et al*., 2011; Harmer *et al*., 2002), and some show high expression during fibre elongation (Shi *et al*., 2006; Yang *et al*., 2014). For instance, a cotton protodermal factor1 gene (*GbPDF1*) is preferentially expressed during fibre initiation by the *HDZIP2ATATHB2* core cis element (Deng *et al*., 2012). However, alpha‐expansins (*GhExp1*) gene highly expressed in developing fibres encodes a cell wall protein and regulates cell wall loosening (Harmer *et al*., 2002). During fibre elongation, many genes related to osmosis regulation are highly expressed. Previously, Ruan *et al*. (2003) reported that antisense suppression of a sucrose synthase (*SuSy*) gene disturbed the fibre elongation, signifying the contribution of *SuSy* in osmosis regulation. In contrast, proline‐rich protein‐coding gene (*GhPRP5*) worked as a negative regulator during cotton fibre development (Xu *et al*., 2013). Cellulose synthesis is a principal event in fibre cells during the secondary cell wall biosynthesis. Previously, many efforts have been made to explore that how the cotton fibre regulates and supports the strong irreversible carbon sink characterized by secondary wall cellulose synthesis (Brill *et al*., 2011; Haigler *et al*., 2007). It has been shown that suppression of *Sus* gene expression affects the cellulose deposition (Ruan, 2007), emphasizing the importance of this enzyme in cellulose synthesis. Subsequently, Brill *et al*. (2011) identified and characterized a novel *Sus* isoform (*SusC*), which was up‐regulated during secondary wall cellulose synthesis in cotton fibre. Besides secondary wall cellulose synthesis, maturation stage of fibre development begins. During fibre maturation, the majority of the expressed genes belong to cellular respiration (Kim *et al*., 2013).

**Table 1:** Functional genomics for fibre traits

Functional study aspect	Specific fibre trait	Cotton species (cultivar)	References
Jasmonate ZIM‐domain protein‐encoding (*GhJAZ2*) gene	Fibre initiation	*G. hirsutum* (TM‐1, YZ1 & Xu142)	Hu *et al*. (2016)
Bulb biogenesis 1 (*GhRBB1_A07*) gene	Fibre quality	*G. hirsutum*	Islam *et al*. (2016b)
Receptor‐like kinase (*RLK*) gene	Fibre strength	*G. hirsutum* (MD52ne & MD90ne)	Islam *et al*. (2016c)
Phytohormone‐related (*PHYA1*) gene	Fibre length	*G. hirsutum* (Coker 312)	Abdurakhmonov *et al*. (2014)
Homeodomain‐leucine zipper (*GhHOX3*) gene	Fibre elongation	*G. hirsutum* (R15), *G. arboreum* (Qinyangxiaozi), *G. herbaceum* & *G. raimondii*	Shan *et al*. (2014)
Calcium sensor (*GhCaM7*) gene	Fibre elongation	*G. hirsutum*	Tang *et al*. (2014)
Brassinosteroid catabolism (*PAG1*) gene	Fibre elongation	*G. hirsutum* (CCRI24)	Yang *et al*. (2014)
LIM domain‐encoding (*WLIM1a*) gene	Fibre elongation and secondary wall synthesis	*G. hirsutum* (R15)	Han *et al*. (2013)
Annexins (*AnnGh3*) gene	Fibre initiation and elongation	*G. hirsutum* (Xuzhou 142, Emian 9,10 & Coker 312)	Li *et al*. (2013a)
Plasma membrane intrinsic protein 2s encoding (*PIP2s*) gene	Fibre elongation	*G. hirsutum* (Xuzhou 142, Emian 9 & Coker 312)	Li *et al*. (2013b)
Proline‐rich proteins (*PRP5*) gene	Fibre length	*G. hirsutum* (Coker 312)	Xu *et al*. (2013)
Protodermal factor1 (*GbPDF1*) gene	Fibre initiation	*G. barbadense* (3‐79) & *G. hirsutum* (Xu142, Xu142 fl & YZ1)	Deng *et al*. (2012)
TCP transcription factor (*GbTCP*) gene	Fibre elongation	*G. barbadense* (3‐79) & *G. hirsutum* (YZ1)	Hao *et al*. (2012)
Sucrose synthase (*GhSusA1*) gene	Fibre length and strength	*G. hirsutum* (TM‐1 & 7235)	Jiang *et al*. (2012)
Homeodomain‐leucine zipper (*GhHD‐1*) gene	Fibre initiation	*G. hirsutum* (Acala Maxxa)	Walford *et al*. (2012)
MADS box protein‐coding (*GhMADS11*) gene	Fibre elongation	*G. hirsutum* (Coker312 & Xuzhou 142)	Li *et al*. (2011)
RAD‐like (*GbRL1*) gene	Fibre initiation	*G. barbadense* (Pima‐90)	Zhang *et al*. (2011a)
Auxin biosynthesis (*iaaM*) gene	Fibre initiation	*G. hirsutum* (Jimian 14)	Zhang *et al*. (2011c)
Xyloglucan endotransglycosylase/hydrolase (*GhXTH*) gene	Fibre elongation	*G. hirsutum* (Coker 312)	Lee *et al*. (2010)
Gibberellin 20‐oxidase (*GhGa20ox1‐3*) gene	Fibre initiation and elongation	*G. hirsutum* (Jimian 14)	Xiao *et al*. (2010)
Peroxidase‐encoding (*GhPOX1*) gene	Fibre elongation	*G. hirsutum* (Xuzhou 142)	Mei *et al*. (2009)
Calcium‐dependent protein kinase (*GhCPK1*) gene	Fibre elongation	*G. hirsutum* (TM‐1)	Huang *et al*. (2008b)
Steroid 5a‐reductase (*GhDET2*) gene	Fibre initiation and elongation	*G. hirsutum* (Jimian 14)	Luo *et al*. (2007)
Ethylene biosynthesis (*ACO*) genes	Fibre elongation	*G. hirsutum* (Xuzhou 142)	Shi *et al*. (2006)
*GhMyb25* and the homeodomain genes	Fibre initiation	*G. hirsutum* (Xu 142 or XZ 142) & six lintless lines	Wu *et al*. (2006)
WDT‐repeat (*GhTTG1‐GhTTG4*) genes	Fibre initiation	Different cotton species	Humphries *et al*. (2005)
Actin cytoskeleton (*ACTIN*) genes	Fibre elongation	*G. hirsutum* (Coker 312)	Li *et al*. (2005)

Many genes encoding transcription factors, that are *MYB*, *C2H2*, *bHLH*, *WRKY* and *HD‐ZIP* families, were also expressed during cotton fibre development. Previously, various studies indicated that *MYB*‐related genes have high expression during fibre development in *G. hirsutum* (Machado *et al*., 2009; Pu *et al*., 2008). For example, expression studies of six *MYB*‐related genes in *G. hirsutum* indicated that *GhMYB6* has high expression in cotton fibre (Loguercio *et al*., 1999), while *R2R3 MYB‐*like transcription factor‐encoding gene ‘*GhMYB109*’ is expressed particularly in fibre initiation and elongation (Suo *et al*., 2003). The *RAD*‐like *GbRL1* was also highly expressed in cotton ovules during fibre initiation (Zhang *et al*., 2011a). *TCP* transcription factor has played a significant role in fibre and root hair development by controlling the jasmonic acid biosynthesis, ethylene signalling, calcium channel and reactive oxygen species (Hao *et al*., 2012). Though, *GhHOX3* from class IV homeodomain‐leucine zipper (*HD‐ZIP*) family showed strong expression during early fibre elongation (Shan *et al*., 2014). Besides transcription factors, phytohormones such as ethylene, auxins and brassinosteroids (BR) also play a critical role during fibre development. Ethylene plays a vital function in fibre elongation by stimulating the pectin biosynthesis network (Qin and Zhu, 2011), while gibberellins (GA) and indole‐3‐acetic acid (IAA) are required for fibre initiation and elongation in cotton (Xiao *et al*., 2010; Zhang *et al*., 2011c). In contrast, the persistent high concentration of jasmonic acid (JA) inhibits fibre elongation (Tan *et al*., 2012).

Although several gene expression studies have been reported on cotton fibre development, some issues are illustrated here. First, most of the differentially expressed genes identified by the comparative analysis are associated with variations between species rather than related to fibre traits. Second, in some cases, the use of the protein‐coding gene sequences from *G. raimondii* and *G. arboreum* may not be accurate enough for gene annotation in tetraploid cotton. Third, it is unknown whether any of the expressed genes recognized from earlier reports had sequence variations between a cotton fibre mutant and its wild type, because only the differentially expressed genes having sequence differences and colocalization with target fibre traits are possible candidates for advanced cotton studies.

## Plant architecture and flowering

The productivity of the cotton plant is mainly affected by the plant architectural traits such as the shape, position of branches and distribution of reproductive structures (Wang *et al*., 2006; Ye and Zhu, 2001). Flowering and terminal loci such as single flower truss (*SFT*) and self‐pruning (*SP*) genes regulate the balance between monopodial and sympodial growth habits in woody perennial plants (Lifschitz *et al*., 2006; McGarry *et al*., 2016; Shalit *et al*., 2009). In cotton, *GhSP* gene is required to maintain both monopodial and sympodial branches and is also vital to ascertain cambial activity (McGarry *et al*., 2016). However, *GhSFT* stimulates the quick onset of sympodial branching and flowering inside the shoots of day neutral and wild photoperiodic accessions (McGarry *et al*., 2016). The floricaula/leafy homologs of cotton also play an important role in the flower initiation, *LFY* (*GhLFY*) gene from *G. hirsutum* was expressed in the shoot apex (Li *et al*., 2013d) with extensive up‐regulation at the third stage of true leaf expansion, and it might function downstream of *MADS* box *GhSOC1* gene.

The time of floral initiation is one of the most important factors related to early maturation of cotton. Many genes have been differentially expressed during floral initiation (Table 2), including those encoding the *B3*, *MADS* and *MYB* domain transcription factors (Wu *et al*., 2015). *MADS* box genes are an important class of transcription factors in plants, involved in various cellular processes particularly in floral developmental processes, that is *GhMADS3* (Guo *et al*., 2007b) and *GhMADS9* (Shao *et al*., 2010). Despite these efforts, little is known about the mechanism underlying plant architecture and floral development in cotton. Nevertheless, it is expected that recent advances in cotton genome sequencing and transformation techniques will increase applications of various molecular biology approaches in cotton, which may help to explore the role of different genes during plant architecture and floral development.

**Table 2:** Functional genomics for plant architecture and flowering

Functional study aspect	Specific trait	Cotton species (cultivar)	References
Late meristem identity1‐D1b* *(*GhLMI1‐D1b*) gene	Leaf shape	*G. hirsutum*	Andres *et al*. (2017)
Single flower truss (*GhSFT*) and self‐pruning (*GhSP*) genes	Monopodial and sympodial branches	*G. hirsutum* (TX701 & DP61)	McGarry *et al*. (2016)
MADs box (*GhSOC1* and *GhMADS42*) genes	Flowering	*G. hirsutum* (CCRI36)	Zhang *et al*. (2016b)
Flowering‐promoting factor 1 (*GhFPF1*) gene	Flowering time control and shade avoidance	*G. hirsutum* (TM1 & CCRI36)	Wang *et al*. (2014a)
Leafy (*GhLFY*) gene	Shoot apex	*G. hirsutum* (CCRI36)	Li *et al*. (2013d)
Florigen‐encoded flowering locus T (*FT*) gene	Determinate growth	*G. hirsutum* (TX701 & DP61)	McGarry *et al*. (2013)
Sepallata (*GhSEP*) gene	Squares or flowers	*G. hirsutum*	Lai *et al*. (2011)
Mitogen‐activated protein kinase (*GhMAPK7*) gene	Plant growth and development	*G. hirsutum* (Lumian 22)	Shi *et al*. (2010)
MADS box (*GhMADS9*) gene	Anther/pollen development	*G. hirsutum* (Coker312)	Shao *et al*. (2010)
MADS box (*GhMADS3*) gene	Stamens and carpels	*G. hirsutum* (Xuzhou 142 & Chuanmian 239)	Guo *et al*. (2007b)

## Abiotic stresses

Cotton's production is limited by various abiotic stresses, which cause about 73% yield loss worldwide (Saranga *et al*., 2009). Among different abiotic stresses, drought and salinity are the two main factors that affect the cotton production and it has become a challenging task to improve tolerance in cotton against these stresses. Previously, few stress‐related genes such as *GhCIPK6* (He *et al*., 2013), *GbRLK* (Zhao *et al*., 2013), *GhMKK1* (Lu *et al*., 2013) and *GhSnRK2* (Bello *et al*., 2014) have been reported in cotton (Table 3). A cotton Raf‐like MAP3K (*GhMAP3K40*) gene positively regulates defence response but mediates reduced tolerance to biotic and abiotic stresses in transgenic *Nicotiana benthamiana* (Chen *et al*., 2015). However, the overexpression of annexin‐encoding (*GhAnn1*) gene showed higher chlorophyll content, increased peroxidase activities and lower lipid peroxidation levels, which ultimately increase the salt and drought stress tolerance in transgenic cotton (Zhang *et al*., 2015a). Previous studies have also reported that CBL‐interacting protein kinase (*GhCIPK6*) and sucrose nonfermenting 1‐related protein kinase 2 (*SnRK2*) genes are also involved in abiotic stress tolerance in cotton (Bello *et al*., 2014; He *et al*., 2013). Genes related to ethylene, abscisic acid and jasmonic acid signalling pathways have also played a significant role in drought tolerance (Chen *et al*., 2013). Further, 1528 and 1128 leaf‐ and root‐related genes with 28 biological pathways have been identified in response to water‐deficient conditions (Ranjan and Sawant, 2015), which signifies that leaves are distinct from roots for molecular mechanisms of drought tolerance in cotton.

**Table 3:** Functional genomics for abiotic stress

Functional study aspect	Abiotic stress	Cotton species (cultivar)	References
ERF‐encoding (*GhERF38*) gene	Salinity, drought and abscisic acid	*G. hirsutum* (Coker 312)	Ma *et al*. (2017)
bZIP‐encoding (*GhABF2*) gene	Drought and salinity	*G. hirsutum* (Simian 3)	Liang *et al*. (2016)
WRKY transcription factor‐encoding (*GhWRKY25*) gene	Drought and salinity	*G. hirsutum* (Lumian 22)	Liu *et al*. (2016b)
Trehalose‐6‐phosphate synthase (*GhTPS11*) gene	Heat, drought, salinity, gibberellin and abscisic acid	*G. hirsutum* (ZM19)	Wang *et al*. (2016a)
NAC domain‐encoding (*GbNAC1*) gene	Abscisic acid, mannitol and NaCl	*G. barbadense* (Xinhai 15 & Xinhai 16)	Wang *et al*. (2016b)
Mitogen‐activated protein kinase (*GhMAP3K40*) gene	Drought and salinity	*G. hirsutum* (Lumian 22)	Chen *et al*. (2015)
WRKY transcription factor‐encoding (GhWRKY41) gene	Drought and salinity	*G. hirsutum* (Lumian 22)	Chu *et al*. (2015)
Annexin gene (*GhAnn1*)	Salinity	*G. hirsutum* (7235)	Zhang *et al*. (2015a)
Sucrose nonfermenting 1‐related protein kinase 2 (*GhSnRK2*) gene	Drought, cold, abscisic acid and salinity	*G. hirsutum* (CCRI24)	Bello *et al*. (2014)
Mitogen‐activated protein kinase (*GbMPK3*) gene	Drought	*G. barbadense* (7124)	Long *et al*. (2014)
WRKY transcription factor (*GhWRKY39‐1*) gene	Salinity	*G. hirsutum* (Lumian 22)	Shi *et al*. (2014a)
WRKY transcription factor‐encoding (*GhWRKY39*) gene	Salinity	*G. hirsutum* (Lumian 22)	Shi *et al*. (2014b)
CBL‐interacting protein kinase (*GhCIPK6*) gene	Salinity, drought and abscisic acid	*G. hirsutum* (YZ‐1)	He *et al*. (2013)
NAC domain protein (*GhNAC7‐GhNAC13*) genes	Cold, abscisic acid, drought and salinity	*G. hirsutum* (Coker 312)	Huang *et al*. (2013)
Mitogen‐activated protein kinase (*GhMPK6a*) gene	Salinity and drought	*G. hirsutum* (Lumian 22)	Li *et al*. (2013e)
Mitogen‐activated protein kinase kinases (*GhMKK1*) gene	Salinity and drought	*G. hirsutum* (Lumian 22)	Lu *et al*. (2013)
Receptor‐like kinase (*GbRLK*) gene	Salinity and drought	*G. barbadense* (Hai 7124)	Zhao *et al*. (2013)
Mitogen‐activated protein kinase (*GhMKK5*) gene	Salinity and drought	*G. hirsutum* (Lumian 22)	Zhang *et al*. (2012)
Mitogen‐activated protein kinase (*GhMPK16*) gene	Drought	*G. hirsutum* (Lumian 22)	Shi *et al*. (2011)
Mitogen‐activated protein kinase (*GhMPK2*) gene	Salinity and drought	*G. hirsutum*	Zhang *et al*. (2011b)
Ethylene responsive (*GhERF2*, *GhERF3*, *GhERF6*) genes	Ethylene, abscisic acid, salt, cold and drought	*G. hirsutum* (Zhongmian 12)	Jin *et al*. (2010)
DRE‐binding transcription factor (*GhDREB*) gene	Drought, salinity and cold	Cotton (Simian 3)	Gao *et al*. (2009)
CCCH‐type zinc finger protein‐encoding (*GhZFP1*) gene	Salinity	*G. hirsutum* (ZMS19)	Guo *et al*. (2009)
CBF/DREB1‐encoding (*GhDREB1*) gene	Freezing, salinity and osmotic	*G. hirsutum*	Huang *et al*. (2009)
NAC transcription factor (*GhNAC1‐GhNAC6*) genes	Drought, salinity, cold and abscisic acid	*G. hirsutum* (Jinmian 19)	Meng *et al*. (2009)
DRE‐binding protein‐encoding (*GhDBP2*) gene	Drought, low temperature and abscisic acid	*G. hirsutum* (Zhongmian 12)	Huang *et al*. (2008a)
Ethylene response factors (*GhERF1*) gene	Ethylene, abscisic acid, salinity, cold and drought	*G. hirsutum* (Zhongmian 12)	Qiao *et al*. (2008)
DREB1/CBF‐like (*GhDREB1L*) gene	Low temperature, drought and salinity	*G. hirsutum* (Zhongmian 35)	Huang *et al*. (2007)

Comparative analysis of genome‐wide expression profile reveals that different genes, transcription factors and physiological processes work together to induce stress tolerance (Ranjan *et al*., 2012). Transcription factors could be used as candidate genes to increase stress tolerance in cotton as they act in response to stress signals by regulating the expression of various downstream genes involved in response to high salt, drought and cold stresses (Guo *et al*., 2015a). The *WRKY* is one of the largest families of transcription factors in plants that bind to particular DNA sequences to repress or activate the transcription of various genes (Dou *et al*., 2014). To date, various *WRKY*‐based studies have been conducted in cotton against abiotic stresses (Shi *et al*., 2014b; Zhou *et al*., 2014). Additionally, *NAC* is also an important class of the transcription factors and its proteins are distinguished by a highly conserved N‐terminal (DNA‐binding) and highly divergent C‐terminal regions (Puranik *et al*., 2011), which is valuable for the diversity in the transcriptional activities. In cotton, putative *NAC* genes ‘*GhNAC1–GhNAC6*’ (Meng *et al*., 2009) and ‘*GhNAC7–GhNAC13*’ (Huang *et al*., 2013) have been highly expressed in leaves and roots and distinctively regulate under high salt, drought, cold and ABA conditions. The basic region leucine zipper (*bZIP*) and ethylene response factors (*ERF*) are among the largest and most diverse transcription factor families involved in stress tolerance in many plant species. However, in cotton, few members of these families, that are *GhABF2* (Liang *et al*., 2016), *GhERF2*, *GhERF3*, *GhERF6* (Jin *et al*., 2010) and *GhERF38* (Ma *et al*., 2017), have been characterized for stress tolerance (Abid *et al*., 2017). Evidence from transgenic plants has demonstrated that C‐repeat/dehydration‐responsive element binding factor (*GhDREB1*) gene could function as positive regulators to enhance abiotic stress tolerance in cotton (Huang *et al*., 2009).

The development of stress tolerance cotton cultivars has become more feasible in recent years; though, it is still a difficult task that needs extensive interdisciplinary research efforts. Wild *Gossypium* species offer genetic diversity related to stress tolerance (Saranga *et al*., 2009), which can be employed in future cotton improvement programmes. More recently, different genomic tools have also become available to identify underlying genes and pathways during stress tolerance and to transfer them into different cotton cultivars.

## Biotic stresses

Globally, biotic stresses such as insects, weeds and diseases occur with different levels of intensity, which may not be relevant in a particular year but they generally reduce plant yield in most years (Fritsche‐Neto and Borem, 2012). Among the different biotic factors, cotton breeding against disease resistance remains the primary objective. The shortage of resistant cotton germplasms makes *Verticillium* wilt the most serious disease to influence cotton production (Chang *et al*., 2008). The molecular mechanisms of resistance to *V. dahliae* reported that cotton phenylpropanoid pathway (Xu *et al*., 2011), terpenoid pathway (Luo *et al*., 2001), salicylic acid, reactive oxygen species and jasmonic acid signalling pathways (Xu *et al*., 2014) are important contributors to the pathogen response (Table 4). Plant mitogen‐activated protein kinase (*MAPK*) cascades have also been shown to regulate a number of stress responses. In cotton, *GhMPK16* from D‐MAPK group has been characterized, which is involved in disease resistance (Shi *et al*., 2011). Additionally, transgenic cotton plants expressing the synthetic antimicrobial peptide (*D4E1*) gene showed the significant resistance to disease and mycotoxin causing fungal pathogens (Rajasekaran *et al*., 2005). Many *WRKY* proteins have also played a regulatory function in response to different pathogen infections either by regulating itself or due to their proximity to well‐characterized genes that play a central role in cotton defence (Zhou *et al*., 2014). Cotton leaf curl virus (*CLCuV*) is also one of the important rising threats to cotton production in different countries. It has been reported that resistance against *CLCuV* is conferred by two dominant and one suppressor gene (Rahman *et al*., 2005). In other studies, antisense coat protein gene (*AV1*) and truncated *AC1* gene were targeted for restricting viral replication and movement in transgenic cotton (Amudha *et al*., 2013; Hashmi *et al*., 2011).

**Table 4:** Functional genomics for biotic stress

Functional study aspect	Biotic stress	Cotton species (cultivar)	References
Jasmonate ZIM‐domain (*GhJAZ2*) gene	*Verticillium dahliae*	*G. hirsutum*	He *et al*. (2017)
*GR79‐EPSPS* and N‐acetyltransferase (*GAT*) genes	Resistant to glyphosate	*G. hirsutum* (*R18*)	Liang *et al*. (2017)
Jasmonate ZIM‐domain interactor (*NINJA*) gene	*Verticillium dahliae*	*G. hirsutum* (BD18)	Wang *et al*. (2017a)
*Ve* homologous (*Gbvdr3*) gene	*Verticillium dahliae*	*G. barbadense* (7124)	Chen *et al*. (2016)
MYB transcription factor (*GhMYB108*) gene	*Verticillium dahliae*	*G. hirsutum* (BD18)	Cheng *et al*. (2016a)
*Tectaria macrodonta* (*Tma12*) gene	Cotton leaf curl virus and whitefly	*G. hirsutum* (Coker 312)	Shukla *et al*. (2016)
NAC transcription factor (*GbNAC1*) gene	*Verticillium dahliae*	*G. barbadense* (Xinhai 15 & Xinhai 16)	Wang *et al*. (2016c)
WRKY transcription factor (*GbWRKY1*) gene	*Botrytis cinerea* and *V. dahliae*	*G. barbadense* (7124) & *G. hirsutum* (YZ1)	Li *et al*. (2014a)
WRKY transcription factor (*GhWRKY39‐1*) gene	*R. solanacearum* and *R. solani*	*G. hirsutum* (Lumian 22)	Shi *et al*. (2014a)
Mitogen‐activated protein kinase (*GhMPK6a*) gene	*Ralstonia solanacearum*	*G. hirsutum* (Lumian 22)	Li *et al*. (2013e)
WRKY transcription factor (*GhWRKY15*) gene	Viral and fungal pathogens	*G. hirsutum* (Lumian 22)	Yu *et al*. (2012)
Mitogen‐activated protein kinase (*GhMKK5*) gene	*Ralstonia solanacearum*	*G. hirsutum* (Lumian 22)	Zhang *et al*. (2012)
Disease resistance (*GhNDR1*) and MAP kinase kinase 2 (*GhMKK2*) genes	*Verticillium dahliae*	*G. hirsutum* (Deltapine 90, R135, Phytogen 480WR, Phytogen 425RF, FM 832, PSC 355 & FM 9160B2F)	Gao *et al*. (2011)
WRKY transcription factor (*GhWRKY3*) gene	*R. solani*, *Colletotrichum gossypii* and *F. oxysporeum*	*G. hirsutum*	Guo *et al*. (2011)
Mitogen‐activated protein kinase (*GhMPK16*) gene	*X. campestris* pv. *malvacearum*, *R. solani* and *C. gossypii*	*G. hirsutum* (Lumian 22)	Shi *et al*. (2011)
Lignin‐related genes	*Verticillium dahliae*	*G. barbadense* (7124) & *G. hirsutum* (YZ‐1)	Xu *et al*. (2011)
Mitogen‐activated protein kinase (*GhMPK7*) gene	*R. solani*, *C. gossypii* and *F*. *oxysporum* f. sp. *vasinfectum*	*G. hirsutum* (Lumian 22)	Shi *et al*. (2010)
CCCH‐type zinc finger protein (*GhZFP1*) gene	*R. solani*	*G. hirsutum* (ZMS19)	Guo *et al*. (2009)
CP4 5‐enolpyruvylshikimate‐3‐phosphate synthase (*CP4 EPSPS*) gene	Resistant to glyphosate	*G. hirsutum* (Coker 312 & Coker130)	Chen *et al*. (2006)
Nonsymbiotic haemoglobin (*GhHb1*) gene	*Verticillium dahliae*	*G. hirsutum* (BD18)	Qu *et al*. (2005)
Synthetic antimicrobial peptide (*D4E1*) gene	*F. verticillioides*, *V. dahlia*, *A. flavus* and *T. basicola*	*G. hirsutum* (Coker 312)	Rajasekaran *et al*. (2005)
(+)‐δ‐Cadinene synthase (*cdn1‐C4*) gene	Bacterial blight	*G. hirsutum*	Townsend *et al*. (2005)

Insect herbivores and cotton plant have waged war from millions of years. In cotton plant, transgenic technology has been mainly used to induce the resistance against insect herbivores. Among the different transgenic approaches against insects, *Cry* gene encoding *Bacillus thuringiensis* toxin has gained a fabulous success against bollworms (Guo *et al*., 2007a; Rashid *et al*., 2008). Recently, *tma‐12* gene encoding insecticidal protein has been identified that gives substantial results against whitefly and cotton leaf curl viral disease (Shukla *et al*., 2016). Many secondary metabolites in cotton such as gossypol and related sesquiterpene aldehydes form phytoalexin chemicals that facilitate it to escape from herbivores. For example, expression of a P450 monooxygenase gene (*CYP6AE14*) is correlated with larval growth and its expression was induced by gossypol (Mao *et al*., 2007, 2011, 2013). Besides insect, cotton yield has also been largely affected by weeds throughout the growing season, which is generally managed by the application of several classes of herbicides. One such herbicide is the glyphosate which has become the most valuable herbicide due to its low cost and broad‐spectrum weed control. Initially, few genetically modified herbicide‐tolerant cotton lines have been developed by transferring gene encoding the 5‐enolpyruvylshikimate‐3‐phosphate synthase isolated from *Agrobacterium* sp. CP4 (*CP4 EPSPS*) (Nida *et al*., 1996). Lately, it was reported that *CP4 EPSPS* gene has played an important role in vegetative tolerance to glyphosate; however, its expression is critical for the development of male reproductive organ in response to high glyphosate application during late developmental stage (Chen *et al*., 2006). Additionally, highly glyphosate‐resistant cotton plants have also been developed by pyramiding the glyphosate resistance and detoxification genes (Liang *et al*., 2017), which presents attractive promise for developing highly herbicide‐resistant cotton cultivars.

## Future perspectives

For determining the entire set of genes with their functions, genome sequencing of an organism is an important prerequisite resource. At present, the sequenced and re‐sequenced genomes of diploid and allotetraploid (Fang *et al*., 2017a,2017b; Li *et al*., 2014b, 2015; Liu *et al*., 2015; Paterson *et al*., 2012; Wang *et al*., 2012; Yuan *et al*., 2015; Zhang *et al*., 2015b) cotton have been available, which presents valuable information for cotton genomes. However, large knowledge gaps still persist as compared to Arabidopsis and rice, concerning with the molecular regulation of the fundamental biological processes. Due to which, characterization and cloning of more essential genes controlling complex traits is a major challenge for current and future cotton functional genomics studies. Currently, there is a dire need to further analyse multiple cotton cultivars which will improve the depth and pave a better way that will lead to more optimized marker applications and automated genotyping platforms for CNV determination (Rasheed *et al*., 2017).

Additionally, the development of a well‐organized system for molecular breeding by various functional components is necessary. Previously, few efforts have been made to develop a multiscale crop system for high‐throughput association studies of composite traits, that is the ePlant model (Zhu *et al*., 2013). Moreover, a revolution is underway in cotton functional genomics which is spearheaded by the CRISPR/Cas9 system due to its several valuable features. Also, there is a need to understand the composite connections among genes related to different cotton traits under control as well as diverse environmental conditions which ultimately boost our capability to adapt cotton plants appropriate for improvement in various traits.

Harnessing the full potential of functional genomics requires a multidisciplinary approach and integrated knowledge of the molecular and other biological processes underlying different traits because gene functions cannot be inferred by only one approach. The addition of information obtained from genomics, transcriptomics, proteomics and epigenomics studies of cotton will help us to critically explore and investigate the different regulatory pathways underlying different traits. Also, new user‐friendly bioinformatics tools and software with better resolving power and technological improvements need to be developed to increase the potential offered by functional genomics. The resulting huge amount of data from different high‐throughput techniques should, in turn, be further organized, stored and interconnected into fundamental timely updated databases in order to let easy extraction and comparison that will increase the understanding and opportunities for future functional genomics advancements in cotton.

## Conclusion

The strong background of cotton genetics and the great efforts of the cotton genome consortium led to the start of cotton genome sequencing in 2007. With the wealth of cotton genome sequence information, cotton genomics research has entered the phase of fast functional characterization of all genes. However, despite great efforts in whole‐genome sequencing and re‐sequencing of cotton, large knowledge gaps still persist as compared to model plants *Arabidopsis* and rice. Therefore, next stage of cotton genomics requires draft genome refinement, re‐sequencing broad diversity panels and diverse wild relatives to better understand its genome. However, for taking the full benefits of the available genomic information on cotton genes, only the multidisciplinary integrated approach allows their functional characterization. So, advances in functional genomics of cotton will depend on developing high‐throughput technologies and integrating multidisciplinary approaches including genomics, transcriptomics, proteomics, epigenomics and bioinformatics in upcoming cotton improvement programmes.

## Conflict of interests

The authors declare that there is no conflict of interests regarding the publication of this manuscript.

## Author contributions

JA and GS conceived and designed the experiments; all authors performed data analysis and interpretation; JA, WM, MAA and GS wrote the manuscript.
